# Synergistic Effects of Artichoke Stem Waste and TiO_2_ Nanoparticles in Additively Manufactured Hybrid Polymer Composites

**DOI:** 10.3390/polym17202740

**Published:** 2025-10-13

**Authors:** Saadet Güler

**Affiliations:** Department of Metallurgical and Materials Engineering, Faculty of Engineering and Architecture, Izmir Katip Celebi University, Izmir 35620, Turkey; saadet.guler@ikcu.edu.tr

**Keywords:** additive manufacturing methods, stereolithography (SLA), artichoke stem waste (ASW), titanium oxide nanoparticles (TiO_2_ NPs), polymer composites

## Abstract

This study addresses the critical need for sustainable material development by thoroughly investigating the synergistic effects of artichoke stem waste (ASW) and titanium dioxide nanoparticles (TiO_2_ NPs) on the properties of epoxy matrix composites. This research uniquely utilizes stereolithography (SLA)-based 3D printing technology for the fabrication and characterization of polymer matrix composites. The study systematically investigates three distinct composite formulations: artichoke stem waste/epoxy, TiO_2_ nanoparticles/epoxy, and a novel hybrid of artichoke stem waste/TiO_2_ nanoparticles/epoxy composites. Each formulation was prepared at three different loading concentrations to determine their optimal performance. The fabricated composites underwent comprehensive characterization, including meticulous evaluations of their mechanical (tensile), thermal (Thermogravimetric Analysis (TGA)), morphological (Scanning Electron Microscopy (SEM)), and chemical-bonding (Fourier Transform Infrared (FT-IR) spectroscopy) properties. Additionally, X-ray Diffraction (XRD) and FT-IR analyses were performed to structurally characterize the raw materials (pristine (cured epoxy), ASW, and TiO_2_ NPs) and the final composite structures. The findings indicate that the incorporation of ASW and TiO_2_ NPs significantly enhances the performance of epoxy composites. This discovery is significant as it demonstrates the successful valorization of agricultural waste into high-performance composite materials and advances the capabilities of 3D printing technology in sustainable materials science. The results of this study offer critical insights, substantially contributing to the development of sustainable and high-value materials.

## 1. Introduction

The global composite materials market faces increasing pressure to develop sustainable alternatives that can effectively compete with synthetic systems while simultaneously addressing environmental concerns. Natural fiber-reinforced polymer composites have emerged as promising solutions, offering biodegradability, renewable sourcing, and a reduced environmental impact compared to conventional systems [1,2]. Agricultural waste materials present exceptional opportunities due to their abundance (exceeding 4 billion tons annually worldwide) and potential to create additional revenue streams for farming communities [3]. Artichoke (*Cynara scolymus* L.) cultivation generates substantial quantities of lignocellulosic stem waste, with global production exceeding 1.8 million tons annually [4,5]. This waste typically contains 48–52% cellulose, 22–28% hemicellulose, and 18–23% lignin, exhibiting a hierarchical fibrous architecture with fiber diameters ranging from 15 to 45 μm and aspect ratios of 60–120 [6,7]. The natural fiber structure exhibits mechanical interlocking potential with polymer matrices through hydrogen bonding mechanisms [8]. The incorporation of titanium dioxide (TiO_2_) nanoparticles into polymer matrices represents a sophisticated approach to developing multifunctional composite materials [9,10,11]. Anatase TiO_2_ exhibits exceptional characteristics including a high elastic modulus (280–300 GPa), superior chemical stability, and unique photocatalytic activity [12,13]. Studies report tensile strength enhancements of 25–45% and elastic modulus improvements of 20–40% in polymer nanocomposites [14].

Digital light processing (DLP) and stereolithography (SLA) technologies have revolutionized composite fabrication by enabling unprecedented precision control over material distribution and geometric complexity [15,16,17]. Specifically, DLP technology achieves layer thicknesses as low as 10 μm and dimensional accuracies within ±15 μm through photopolymerization processes [18,19]. Recent work by Kılınç and colleagues has significantly advanced the understanding of parameter optimization in DLP-based manufacturing, demonstrating critical relationships between processing parameters and mechanical properties [20,21,22,23]. Hybrid composite systems combining natural fibers with inorganic nanoparticles represent an emerging frontier, potentially yielding synergistic property enhancements that surpass individual reinforcement contributions [24]. Recent investigations demonstrate that optimally designed hybrid systems can achieve tensile strength enhancements of 45–70%, flexural modulus increases of 30–55%, and thermal stability improvements of 25–40 °C [25,26]. Despite these significant advances, critical knowledge gaps persist, including an insufficient understanding of synergistic reinforcement mechanisms, a lack of systematic optimization protocols for DLP-SLA processing, and limited comprehensive characterization of interfacial bonding mechanisms [27,28,29,30]. These deficits impede the translation of research into industrial applications [31].

The present investigation addresses these gaps through a comprehensive exploration of the synergistic effects between ASW and TiO_2_ NPs in epoxy-based composites manufactured via DLP-SLA technology. This research contributes to sustainable materials science by establishing predictive structure–property relationships and demonstrating the transformation of agricultural waste into high-performance materials.

## 2. Materials and Methods

### 2.1. Materials

Epoxy resin (AnyCubic Technology Co., Ltd., Hong Kong/China) was obtained from a commercial source. ASW was sourced from local agricultural providers. The unprocessed artichoke stems were meticulously cleansed to eliminate contaminants, dehydrated in an oven at 80 °C for 24 h, and subsequently pulverized into a fine powder utilizing a blade mill. The resultant powder was sieved by using 200 mesh (75 µm) stainless steel sieve, and particles less than 75 microns were utilized for composite fabrication. Nano titanium dioxide (TiO_2_) powder, with average particle size of 25–35 nm, density of approximately 4.23 g/cm^3^, and purity of 99.5 (Nanokar Kimyevi Maddeler San. ve Tic. Ltd. Şti., Istanbul/Türkiye), was acquired through commercial means.

### 2.2. Composite Preparation

Three unique varieties of polymer matrix composites were synthesized based on the formula outlined in Table 1. Each mixture was formulated with three distinct filler loading ratios to examine the influence of reinforcing concentration on composite characteristics.

**Figure 1:**
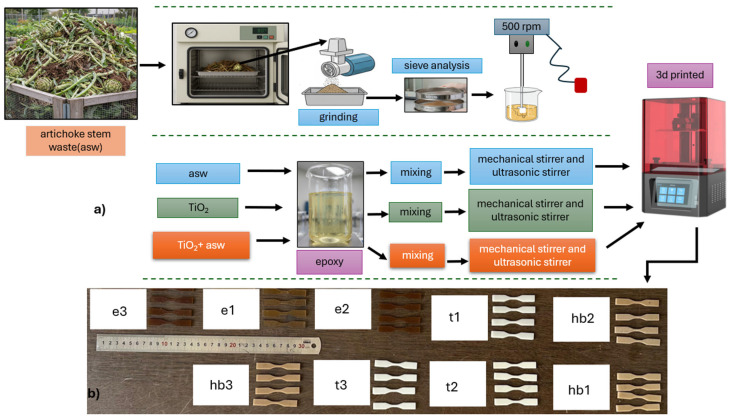
(**a**) Fabrication process of the composite materials and (**b**) 3D-printed tensile specimens of the produced composites.

The mixing procedure was carried out at room temperature under controlled laboratory conditions to ensure uniform dispersion of the fillers within the resin matrix. Initially, the components were blended using a mechanical stirrer at 500 rpm for 30 min to achieve preliminary particle dispersion. This step was followed by an additional 30 min of ultrasonic treatment (35 kHz, 200 W, at room temperature), which effectively minimized particle agglomeration and improved the overall homogeneity of the suspension. Three-dimensional CAD models of the specimens were created in SolidWorks (2021 Student edition) and exported in STL format. The slicing process was performed using HALOT BOX, a resin slicing software specifically optimized for LCD-based printing systems, to generate the G-code. The homogenized suspensions were then processed via additive manufacturing, employing a Creality Halot-One CL-60 digital light processing (DLP) 3D printer. The entire production process is shown in Figure 1. Detailed printing parameters are provided in Table 2.

After printing, all samples were immersed in isopropyl alcohol (IPA) and subjected to gentle agitation for 15 min to remove uncured resin residues. Finally, post-curing was applied using UV source light for 60 min to complete photopolymerization and stabilize the composite structure.

### 2.3. Characterization

The morphological, crystallographic, and chemical structures of the as-received TiO_2_ and ASW powders used in this study were meticulously investigated. The morphologies of the powders were analyzed using a scanning electron microscope (SEM) with an accelerating voltage of 5 kV. The crystalline phase structure of the powders was determined by an X-ray diffractometer (Rigaku D/D/max-2200 PC, Rigaku Co. Ltd., Tokyo, Japan) using Cu-Kα radiation (1.5406 Å) with a scanning rate of 5 °/min in the range of 15° ≤ 2θ ≤ 80°. Finally, functional groups of the powders were identified by Fourier Transform Infrared spectroscopy (FT-IR; Thermo Scientific, Nicolet iS10, Madison, WI, USA) in the wavenumber range of 4000–650 cm^−1^, with a resolution of 2 cm^−1^ and 25 scans per sample. To comprehensively evaluate the structural, thermal, chemical, and mechanical characteristics of the fabricated composites, a series of experimental analyses were conducted. The surface morphologies of ASW and TiO_2_ nanoparticles as well as the produced composites were investigated by scanning electron microscopy (SEM, Zeiss EVO, Oberkochen, Germany). For imaging, nano powders were analyzed at an accelerating voltage of 5 kV, while 3D-printed composite specimens were examined at 15 kV. Prior to observation, all samples were sputter-coated with a thin layer of Au–Pd to improve conductivity. Thermal behavior, including degradation characteristics, was assessed using thermogravimetric analysis (TGA, PerkinElmer STA8000, Waltham, MA, USA). Samples were heated under a nitrogen atmosphere from room temperature to 550 °C at a constant heating rate of 10 °C/min. The chemical bonding states of both neat and reinforced composites were examined through Fourier Transform Infrared spectroscopy (FTIR; Thermo Scientific Nicolet iS10). Measurements were performed using an attenuated total reflectance (ATR) unit, and spectra were acquired in the range of 4000–650 cm^−1^ with 25 scans per sample, presented in transmittance mode. Mechanical properties were evaluated by tensile testing. Tensile tests were performed with three replicates by using Zwick Roell/Z250 (Zwick Roell, Ulm, Germany) universal testing machine at a crosshead speed of 2 mm/min in accordance with ASTM D638, providing tensile strength values.

## 3. Results and Discussion

### 3.1. Characterization of Raw Materials

**Figure 2:**
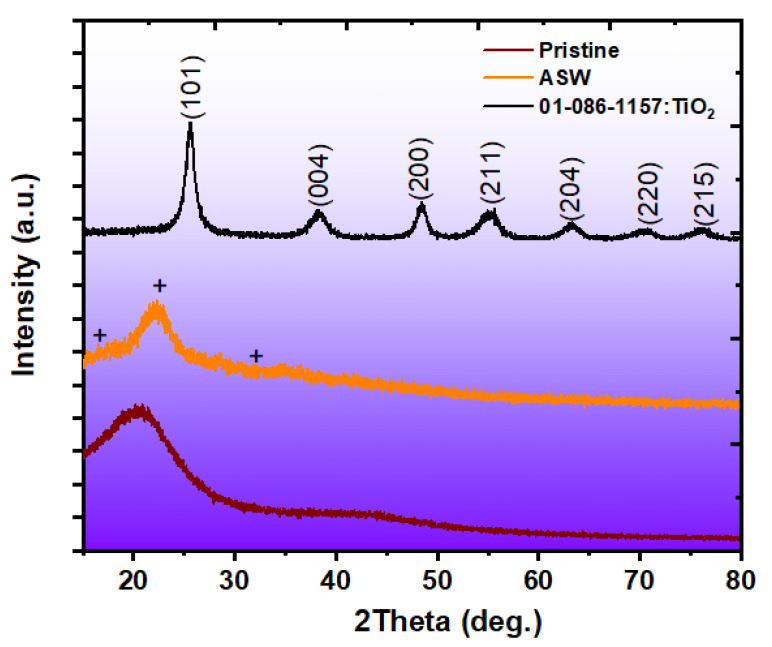
X-ray Diffraction (XRD) patterns of pristine (cured epoxy), artichoke stem waste (ASW), and TiO_2_ nanoparticle samples.

Figure 2 shows the X-ray Diffraction (XRD) patterns of the pristine (cured epoxy resin), ASW, and TiO_2_ NP samples. The pristine samples exhibit a broad amorphous hump centered around 20–25° (2θ), indicating the absence of long-range crystalline order that is typical of crosslinked thermoset polymers.

The ASW pattern displays characteristic diffraction peaks consistent with its lignocellulosic nature [32]. The prominent peaks observed at 2θ values of approximately 16.2°, 22.6°, and 34.8° correspond to the (101), (002), and (040) crystallographic planes of cellulose, respectively, thereby confirming the presence of crystalline cellulose as the predominant structural component [33,34]. Meanwhile, the TiO_2_ nanoparticle pattern reveals a predominantly anatase phase structure with excellent crystallinity [35]. The diffractogram exhibits well-resolved peaks at 2θ values of 25.3°, 37.8°, 48.0°, 54.0°, 55.1°, 62.7°, 68.8°, and 75.0°, corresponding to the (101), (004), (200), (211), (204), (220), (215), and (224) crystallographic planes of anatase TiO_2_, respectively [36].

**Figure 3:**
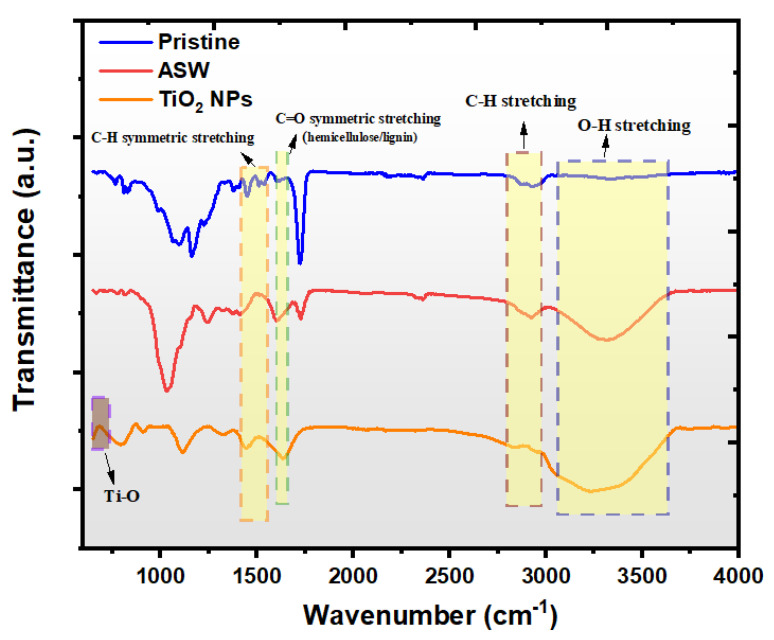
FT-IR spectra of the pristine (cured epoxy), ASW and TiO_2_ NPs.

Figure 3 presents the Fourier Transform Infrared (FT-IR) spectra of raw materials, providing detailed information about the chemical functional groups and molecular structure of the pristine (cured epoxy), ASW, and TiO_2_ NP samples. For the ASW component, the broad absorption band in the region of 3200–3400 cm^−1^ is attributed to O-H stretching vibrations from hydroxyl groups present in cellulose, hemicellulose, and lignin components [37]. The absorption bands observed in the 2850–3000 cm^−1^ region correspond to C-H stretching vibrations from methyl and methylene groups present throughout the lignocellulosic structure [38]. The peak at approximately 1730 cm^−1^ is assigned to C=O stretching vibrations from acetyl groups in hemicellulose and ester linkages in lignin. For the TiO_2_ nanoparticles, the characteristic absorption band in the nearly 500–700 cm^−1^ region corresponds to Ti-O lattice vibrations, confirming the presence of the titanium dioxide phase.

**Figure 4:**
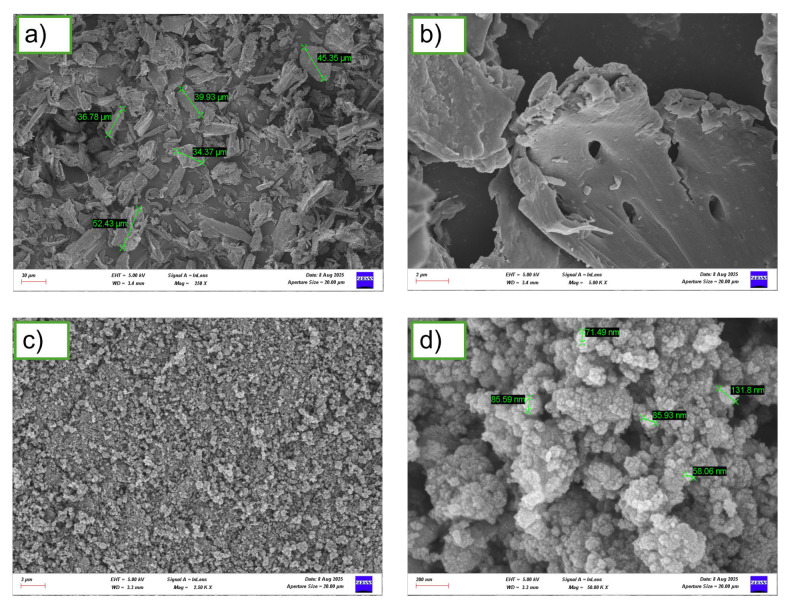
SEM images of (**a**,**b**) ASW and (**c**,**d**) TiO_2_ NPs.

Figure 4 displays SEM images of ASW and TiO_2_ NPs micrographs that enable a comprehensive morphological characterization of the reinforcing phases, elucidating their size distribution, surface properties, and potential for mechanical interlocking within the composite matrix. Figure 4a,b illustrate the morphological features of ASW particles, revealing the characteristic fibrous and heterogeneous structure typical of lignocellulosic materials [39]. These particles display variable forms with rough, textured surfaces, which are advantageous for mechanical interaction with the polymer matrix [40] The reported particle sizes, between 30 and 60 μm, are within an ideal range for composite reinforcement.

Figure 4c,d present the morphological characteristics of TiO_2_ NPs, revealing predominantly spherical to quasi-spherical particle geometries with a relatively uniform size distribution. The individual particle sizes, ranging from 30 to 120 nm, are consistent with the nano-scale dimensions expected for effective reinforcement in polymer nanocomposites [41].

### 3.2. Characterization of Composites

#### SEM Analysis

**Figure 5:**
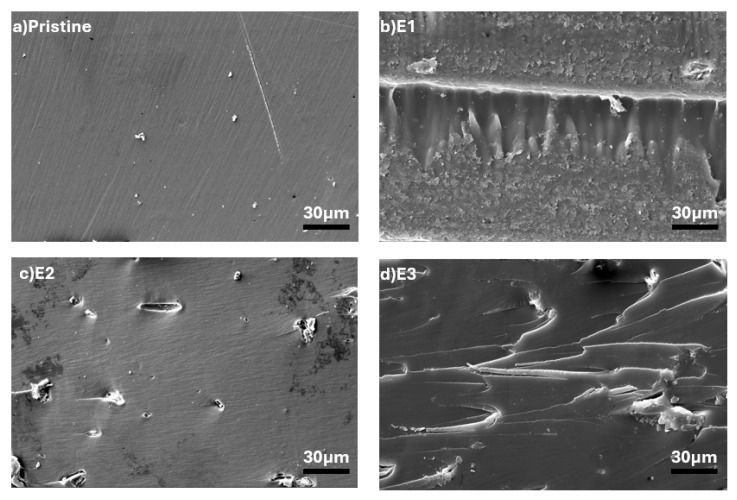
SEM images of (**a**) pristine (cured epoxy) and (**b**–**d**) ASW-reinforced composites.

Figure 5 presents a comprehensive morphological analysis of ASW-reinforced epoxy composites across different loading concentrations (E1, E2, and E3), providing crucial insights into the fiber–matrix interfacial characteristics and dispersion quality. At the lowest loading level E1, the SEM images demonstrate relatively good fiber dispersion within the epoxy matrix, with individual ASW particles being well-separated and embedded within the continuous polymer phase [42]. As the ASW content increases to 2 wt% (E2), the SEM analysis reveals a more complex microstructural arrangement with increased fiber–fiber interactions and the beginning of localized clustering [43]. At the highest loading level (E3), the SEM micrographs reveal significant changes in the composite microstructure, including increased fiber clustering and the formation of fiber-rich regions [44].

**Figure 6:**
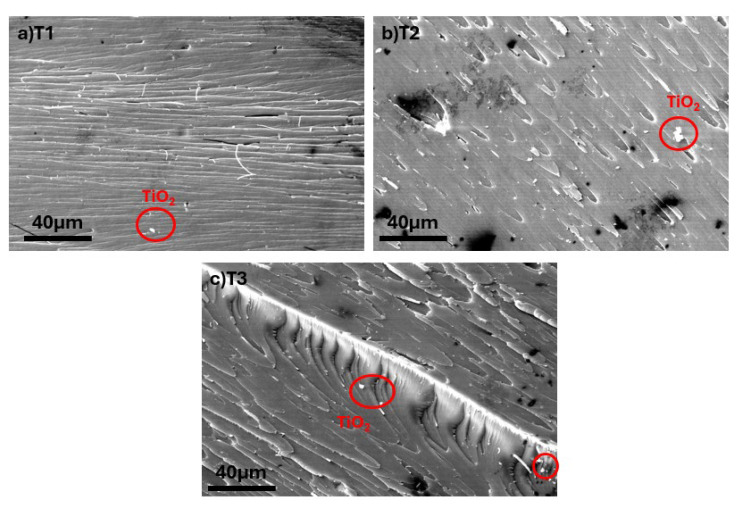
SEM images of (**a**–**c**) TiO_2_ NPs-reinforced composites.

Figure 6 illustrates the morphological characteristics of TiO_2_ NP-reinforced epoxy composites (T1, T2, and T3), demonstrating the critical importance of nanoparticle dispersion quality on composite microstructure and performance [45,46]. The T1 composite (0.5 wt% TiO_2_) exhibits relatively uniform nanoparticle distribution with minimal agglomeration. As the TiO_2_ content increases to 1.0 wt% (T2), the SEM analysis reveals increased agglomeration tendency with the formation of larger nanoparticle clusters [47]. The T3 composite (1.5 wt% TiO_2_) tends to exhibit some degree of agglomeration, which is a common challenge at higher nanoparticle loadings [48].

**Figure 7:**
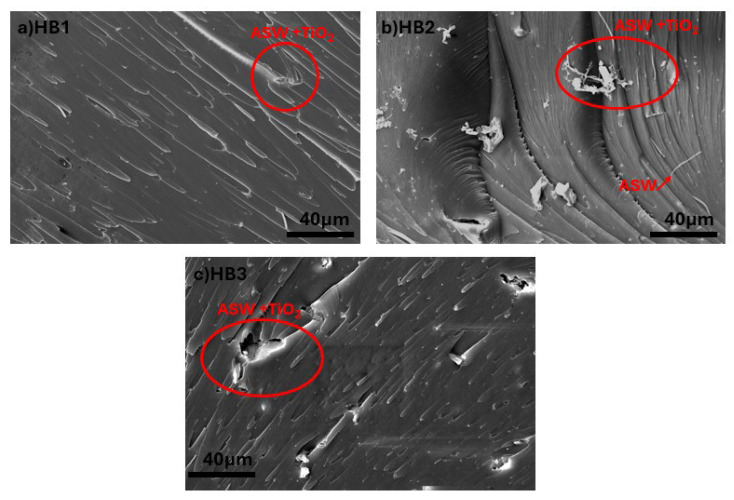
SEM images of (**a**–**c**) ASW/TiO_2_-reinforced (hybrid) composites.

Figure 7 illustrates the morphological characterization of hybrid composites including ASW and TiO_2_ NPs (HB1, HB2, and HB3), highlighting the intricate interactions among various reinforcing elements inside the epoxy matrix [49].

### 3.3. Thermogravimetric Analysis

**Figure 8:**
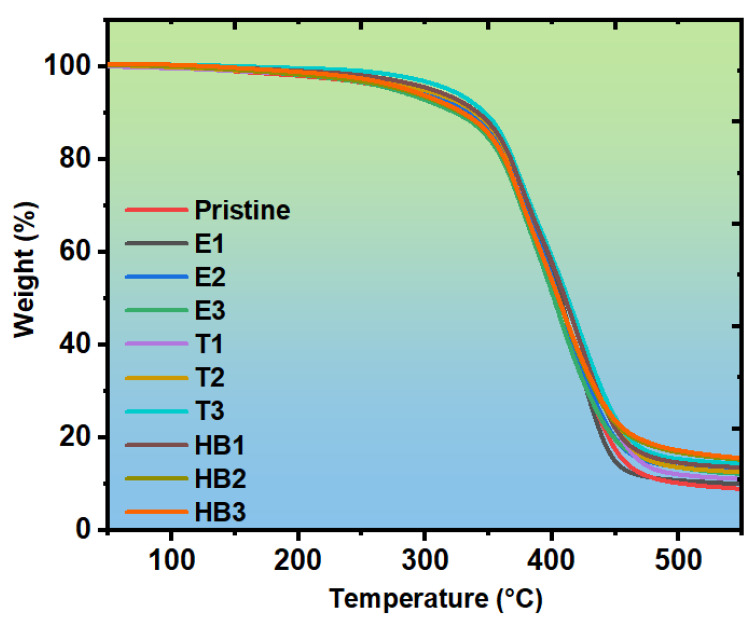
TGA curves of pristine (cured epoxy) and reinforced composites.

Figure 8 displays the thermogravimetric analysis (TGA) results for the pristine samples and all composite formulations, providing detailed insights into the thermal stability and degradation behavior of the reinforced epoxy systems. The corresponding onset degradation temperatures (Tonset) and residual char yields at 550 °C are summarized in Table 3. The Tonset for these composites ranges from approximately 285 °C to 309 °C, a range consistent with the thermal decomposition characteristics of the epoxy matrix and the hemicellulosic fraction from the artichoke stem waste. The samples reinforced with TiO_2_ NPs (T-series) exhibit Tonset values between 298 °C and 305 °C, suggesting that the presence of TiO_2_ contributes to delaying the onset of thermal degradation. The barrier effect provided by TiO_2_ nanoparticles plays a crucial role in impeding the diffusion of degradation products, thereby enhancing thermal stability by reducing the rate of thermal decomposition [50].

The hybrid composites (HB1, HB2, and HB3) demonstrate the most favorable thermal behavior, combining the benefits of both reinforcing phases [51,52]. Their Tonset values range from 304 to 305 °C, representing intermediate stability between the ASW-only and TiO_2_-only systems, indicating that the synergistic interaction between natural fibers and nanoparticles enhances the overall thermal resistance of the composites [53]. This thermal behavior indicates that the combination of natural fibers and inorganic nanoparticles creates a multi-layered thermal protection system that is more effective than either reinforcement alone. The hybrid filler system enables complementary thermal protection mechanisms, where micro-scale ASW fibers provide char formation and thermal insulation, while nano-scale TiO_2_ particles act as thermal barriers and heat sinks, resulting in synergistic thermal enhancement that exceeds the additive contributions of individual components [54].

### 3.4. FT-IR Analysis

**Figure 9:**
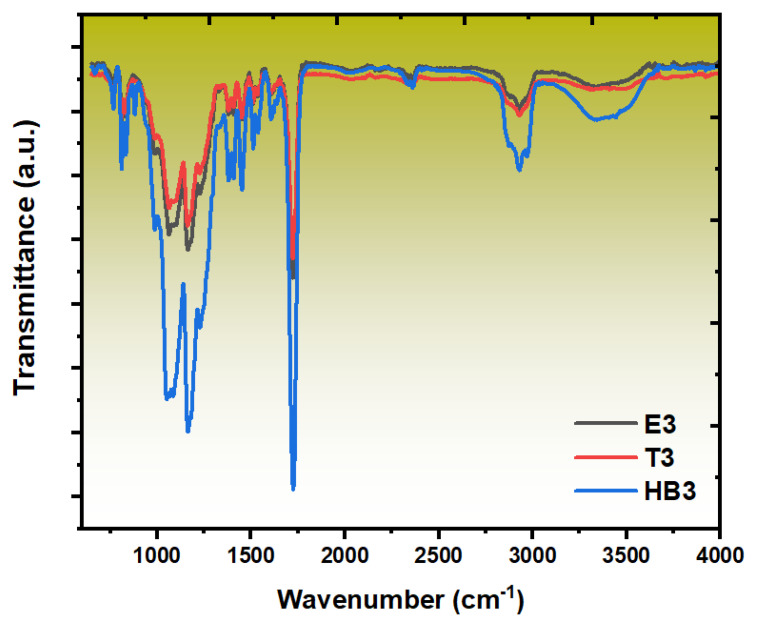
FT-IR spectra of the reinforced composites.

Figure 9 shows the FT-IR spectra of E3, T3, and HB3 composites. The E3 composite spectrum displays distinctive peaks indicative of the lignocellulosic components embedded within the crosslinked epoxy matrix. A notable broad absorption band in the 3200–3600 cm^−1^ range confirms the presence of hydroxyl groups linked to both cellulose and hemicellulose from ASW, as well as residual hydroxyl groups from the epoxy resin itself. These observations align with findings by Sahoo et al. [55], which emphasize the role of hydroxyl groups in forming hydrogen bonds between lignocellulosic fibers and epoxy resins, thereby enhancing adhesion and mechanical properties of composites. Similarly, Karumuri et al. [56] emphasize the significance of hydrogen bond interactions in epoxy–lignocellulose composites, highlighting their influence on the structural integrity and performance of thermoset materials.

The T3 composite spectrum reveals the successful incorporation of TiO_2_ nanoparticles within the epoxy matrix, evidenced by distinct spectroscopic modifications. Effective nanoparticle dispersion within the crosslinked thermoset network is suggested by the emergence of Ti-O stretching vibrations in the nearly 500–700 cm^−1^ range [57]. More importantly, the epoxy characteristic peaks exhibit minor but considerable alterations, especially in the C-O stretching region (1000–1300 cm^−1^), suggesting possible interactions between the epoxy matrix and the hydroxyl groups on the TiO_2_ surface during the crosslinking process.

The HB3 hybrid composite spectrum demonstrates the most complex spectroscopic profile, combining features from both reinforcement phases while exhibiting unique characteristics that suggest synergistic chemical interactions. The hydroxyl region (3200–3600 cm^−1^) shows a broader and more intense absorption compared to individual systems, indicating enhanced hydrogen bonding networks facilitated by the simultaneous presence of cellulosic hydroxyl groups and TiO_2_ surface hydroxyls within the epoxy matrix. This enhanced hydrogen bonding network likely contributes to the improved interfacial adhesion and stress transfer efficiency observed in mechanical testing. The C-H stretching region maintains the characteristic epoxy signatures while incorporating contributions from the natural fiber components, demonstrating successful integration of all phases within the thermoset system.

### 3.5. Tensile Test

**Figure 10:**
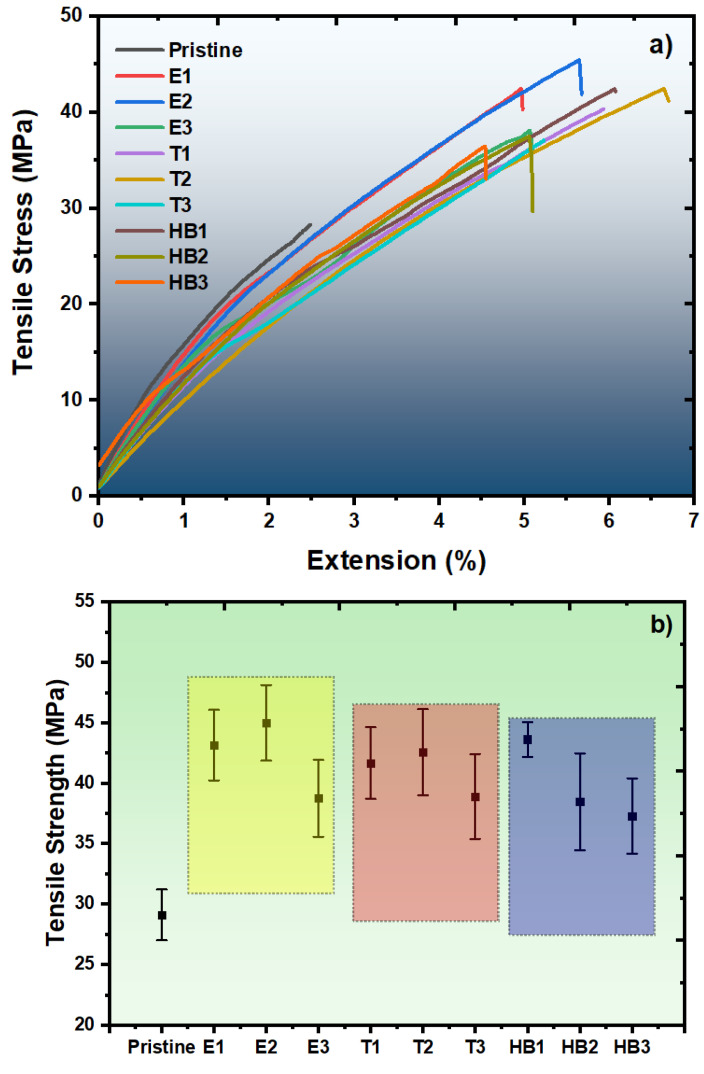
(**a**) Representative tensile curves and (**b**) scatter tensile strength for all samples.

Figure 10a presents representative tensile curves and Figure 10b presents scatter chart composites revealing distinct values across all reinforced composites. As seen in the results, pristine (cured epoxy) samples showed the lowest tensile strength (29.1 MPa). The ASW series demonstrates characteristic optimization behavior, with E2 (44.99 MPa) outperforming both E1 (43.15 MPa) and E3 (38.75 MPa), confirming optimal loading principles observed in recent natural fiber–epoxy studies [58,59].

The TiO_2_ nanoparticle series exhibited a relatively broad optimal loading range, with T1 (41.67 MPa) and T2 (42.56 MPa) demonstrating comparable tensile strengths. A decline observed at T3 (38.91 MPa) suggests that effective dispersion was maintained up to moderate loadings, beyond which particle agglomeration likely diminished reinforcement efficiency.

The hybrid composite systems display various performance characteristics, with HB1 recording the highest tensile strength among the hybrid formulations (43.63 MPa), indicating partial reinforcement synergy [60]. However, none of the hybrid composites exceed the performance of optimal single-phase systems, with HB2 (38.46 MPa) and HB3 (37.29 MPa) showing progressive performance degradation. This trend aligns with contemporary hybrid composite research, indicating that synergistic effects require specific processing optimization to overcome the complexity of multi-phase interactions [61]. The performance levels achieved are similar to those reported in the composites literature, with natural fiber systems typically reaching 35–50 MPa and TiO_2_ nanocomposites reaching 30–45 MPa [62].

## 4. Conclusions

This study demonstrated the successful development of epoxy-based composites reinforced with artichoke stem waste (ASW) and TiO_2_ NPs via stereolithography (SLA) additive manufacturing. Comprehensive characterization and mechanical evaluation revealed distinct optimization patterns and provided valuable insights into multi-scale reinforcement strategies for sustainable composite design. The results confirmed that ASW reinforcement significantly enhanced tensile performance, achieving a maximum strength of 44.99 MPa at 2 wt.% loading (E2), representing an improvement of nearly 55% over pristine(cured epoxy). TiO_2_ NPs also improved performance, with an optimum at 1 wt.% (T2, 42.56 MPa), while higher loadings reduced efficiency due to agglomeration.

The hybrid composites (HB-series) showed complex behavior, with HB1 (43.63 MPa) showing partial reinforcement synergy and stable mechanical performance. Thermal stability analysis (TGA) revealed that hybrid composites (HB2 and HB3) achieved the highest char residues (nearly 12%), demonstrating the complementary effects of lignocellulosic char formation and TiO_2_’s barrier role. FT-IR confirmed hydrogen bonding interactions between ASW and epoxy as well as Ti-O vibrations from TiO_2_, indicating good interfacial integration. SEM analyses supported these findings, highlighting improved filler–matrix adhesion in optimized formulations.

Overall, this research makes a significant contribution to the sustainable valorization of agricultural waste by demonstrating the successful fabrication of high-performance, eco-friendly composites through stereolithography (SLA)-based additive manufacturing. The synergistic incorporation of artichoke stem waste (ASW) and TiO_2_ nanoparticles not only enhanced the tensile and thermal properties but also revealed valuable insights into hybrid reinforcement mechanisms. These findings emphasize the potential of ASW/TiO_2_ systems for developing next-generation lightweight structural and functional components, promoting energy efficiency, durability, and sustainability in advanced polymer composites.

## Figures and Tables

**Table 1:** Sample codes according to the contents of epoxy resin, ASW, and TiO_2_ NPs.

Sample Code	Epoxy Resin (wt%)	ASW (wt%)	TiO_2_ NPs (wt%)
Pristine (cured epoxy)	100	-	-
E1	99.0	1.0	-
E2	98.0	2.0	-
E3	97.0	3.0	-
T1	99.5	-	0.5
T2	99.0	-	1.0
T3	98.5	-	1.5
HB1	98.5	1.0	0.5
HB2	97.5	2.0	0.5
HB3	96.5	3.0	0.5

**Table 2:** SLA printing parameters.

Parameters	Value
Initial Exposure	70 s
Exposure Time	10 s
Motor Speed	1 mm/s
Turn Off Delay	4 s
Rising Height	6 mm
Bottom Exp	10 layers
Layer Thickness	10 micrometer

**Table 3:** TGA data of pristine (cured epoxy) and reinforced epoxy composites.

Sample Name	Onset Temp. (°C)	Residual Weight (%)
Pristine	285 ± 1.1	7.5 ± 0.3
E1	290 ± 1.1	8.4 ± 0.2
E2	292 ± 1.0	8.2 ± 0.3
E3	295 ± 1.3	8.6 ± 0.4
T1	298 ± 1.4	10.4 ± 0.4
T2	300 ± 1.5	11 ± 0.3
T3	305 ± 0.9	11.6 ± 0.5
HB1	304 ± 0.8	11.2 ± 0.3
HB2	307 ± 1.1	11.9 ± 0.2
HB3	309 ± 1.4	12.4 ± 0.4

## Data Availability

The original contributions presented in this study are included in the article. Further inquiries can be directed to the corresponding author.
